# The role of the CCL22-CCR4 axis in the metastasis of gastric cancer cells into omental milky spots

**DOI:** 10.1186/s12967-014-0267-1

**Published:** 2014-09-24

**Authors:** Liang Cao, Xiang Hu, Jian Zhang, Gang Huang, Yi Zhang

**Affiliations:** Department of General Surgery, The First Affiliated Hospital, Dalian Medical University, No.222 Zhongshan road, Dalian, Liaoning China; Plastic and Cosmetic Medical College, Dalian Medical University, 9 West section, Lvshun south road, Dalian, Liaoning 116044 China

**Keywords:** Gastric cancer, Omental milky spot, Peritoneal metastasis, CCL22, CCR4

## Abstract

**Background:**

The omentum is one of the initial sites for peritoneal metastasis because it possesses milky spots that provide a microenvironment for cancer cells to readily migrate and grow into micrometastases. This study investigated the role of the CCL22-CCR4 axis in gastric cancer cells selectively infiltrating into milky spots.

**Methods:**

Gastric cancer MFC cells labelled with DiI were injected intraperitoneally into strain 615 mice. The mice were euthanised at specified intervals and the omentum was excised for immunohistochemistry. The effects of CCL22 on the proliferation and migration of MFCs were assessed by MTT and trans-well assays. RT-PCR and Western blot analysis detected CCR4 mRNA and protein expression levels in MFCs. Immunohistochemistry was used to analyse CCL22 and CCR4 expression in the milky spot micrometastases.

**Results:**

Two weeks after intraperitoneal injection, the milky spot areas were completely occupied by proliferating gastric cancer cells and cell cluster-type micrometastases were observed. In contrast, cancer cells formed single cell-type micrometastases in the non-milky spot areas. MFCs expressed CCR4, which was localised on the cell surface and or in the cytoplasm. Different concentrations of CCL22 significantly increased the proliferation ability of MFCs. Additionally, concentrations of CCL22 between 10–100 ng/ml significantly increased the migration of MFCs. Within omental milky spots, CCL22 was localised mainly on the cell surface and or in the cytoplasm. Within sections of omental milky spot micrometastases, CCR4 was recognised on or in gastric cancer cells, constituent cells milky spots, blood cells and blood endothelial cells.

**Conclusions:**

Omental milky spots are a congenial microenvironment for peritoneal free gastric cancer cells to migrate, survive, and establish cell cluster-type metastases. The CCL22-CCR4 axis contributes to this selective infiltration process.

## Introduction

Peritoneal metastasis is a common pattern of distant metastases in gastric cancer. Numerous studies have confirmed that the prognosis of gastric cancer patients with peritoneal metastasis is poor, even in those patients treated with radical surgery [1]. Peritoneal metastasis develops from micrometastases originating from free peritoneal cancer cells [2]. Therefore, it is important to understand the characteristics and mechanisms involved in the formation of peritoneal micrometastases. Such an understanding will aid in the prevention and recurrence of peritoneal metastases, thus improving the prognosis of gastric cancer patients.

Milky spots are primitive lymphoid tissues in the peritoneal cavity of humans and animals, which exists primarily in the greater omentum. By contrast, relatively few milky spots are found in the mesenterium and pelvic floor, and no milky spots are found in other areas of the peritoneum [3]. Milky spots comprise numerous macrophage and lymphocyte aggregations and are involved in the clearance of particles, bacteria and tumour cells from the peritoneal cavity, playing an important role in peritoneal defence [4-6]. In particular, omental macrophages were found to be cytotoxic against tumour cells [7,8]. Some studies have demonstrated that omental milky spots are well-known sites of metastases of carcinomas of the ovaries, stomach and colon [9]. Cancer cells selectively infiltrate into the milky spots in the early stages of peritoneal cancer dissemination and locate a microenvironment in which they are able to survive, grow and form solid metastases [10]. This preferential attachment can be explained by the existence of certain special characteristics of milky spots, including higher levels of cellular adhesion molecules and growth-stimulatory factors [11,12]. Therefore, while milky spots have cytotoxic properties against tumour cells and play an important role in peritoneal defence, they also provide sites where tumour cell proliferation and micrometastasis occur.

Chemokines are small secreted proteins classified into the four subfamilies based on the sequence of conserved N-terminal cysteine residues: CXC, CC, C, and CX3C [13,14]. Many studies have proved that chemokines and chemokine receptors are causally involved in the metastasis of cancer [15-21]. The macrophage-derived chemokine MDC/CCL22 is one of the CC chemokines produced by macrophages and CCR4 was identified as its specific receptor [17-21], which is also the functional receptor for other CC chemokines. The expression of chemokine receptors is known to be associated with cancer metastases, such as CXCR4, CCR7 and CCR10 in breast cancer [16] and CXCR5, CCR6, CCR7, and CCR4 in pancreatic, gastric and prostate cancers [22,23]. Some studies definitively demonstrated that CXCR4 and CXCL12 play an important role in the metastasis of gastric cancer cells into the peritoneal cavity [23-25].

Omental milky spots comprise numerous macrophages, but the role of the MDC/CCL22-CCR4 axis in gastric cancer cells selectively infiltrating into milky spots has not yet been identified. We therefore examined the expression of MDC/CCL22 and CCR4 in milky spot micrometastases, with the aim of establishing a new treatment method for preventing peritoneal metastasis by focusing on the chemotaxis of gastric cancer cells.

## Materials and methods

### Tumour cell line and animals

Murine gastric cancer MFCs, derived from the strain 615 murine carcinoma of the proximal stomach, were obtained from the central laboratory of the Frist Affiliated Hospital of Dalian Medical University. MFCs were cultured in RPMI-1640 (Gibco) supplemented with 10% foetal bovine serum (Sigma) in an incubator of 5% CO_2_ at 37°C. Once confluent, the cells were trypsinised and rinsed in D-Hanks’ media. A viable cell count was performed using trypan blue exclusion.

Six-week-old strain 615 mice were obtained from the Dalian Medical university of China. The mice were maintained under standard laboratory conditions and had free access to standard laboratory food and water. Study protocols were approved by the Committee for Animal Research of the Dalian Medical university of China according to national guidelines (Permit Number: SYXK2008-0002). All measures were taken to minimise any pain or discomfort.

### Scanning electron microscopy

Omental samples were collected, fixed overnight with 2.5% glutaraldehyde in 0.1 M PIPES buffer (pH 6.9), washed three times in fresh Pipes buffer (pH 6.9), and post-fixed for 1.5 hours in 1% osmium tetroxide in 0.1 M Pipes buffer (pH 6.9). The samples were washed in dH_2_O three times and then dehydrated in increasing concentrations of ethanol (50, 70, 90 and 100%). Specimens were critical point-dried from liquid carbon dioxide, coated to a thickness of 3 nm with an osmium plasma coater, and observed with a Hitachi S-520 scanning electron microscope.

### Transmission electron microscopy

Six omental fat bands obtained from three 615 mice of each gender were used. The bands were immersed in a fixation solution containing 2% glutaraldehyde for 2 hours, then post-fixed in a solution containing 2% osmium tetroxide and 1.5% sucrose in a 0.05 M phosphate buffer for 2 hours at 4°C. The specimens were dehydrated in a graded series of ethanol. After substituting acetone for ethanol, the specimens were embedded in epoxy resin blocks. Ultra-thin sections were stained with uranyl acetate and lead citrate and observed with a transmission electron microscope (JEM-2000EX).

### Tumour cell attachment to the omentum

The MFC cells were incubated in complete RPMI-1640 at a concentration of 2 mg/L DiI (Sigma) for 60 min at 37°C. Cells were washed three times with Hanks’ balanced salt solution. The cell-labelling rate was 100% and 1×10^4^ cells were injected intraperitoneally. The mice were euthanised at 4, 12, 36, 48, 72 and 120 hours, and 7, 10, and 14 days after intraperitoneal injection. The omentum was excised and stretched on microscope slides for further processing.

### HE and immunohistochemical staining

Omental samples were collected, fixed in a 4% formaldehyde solution (pH 7.4) at 4°C for 24 hours, rinsed three times with phosphate buffered saline (pH 7.4) for 3 hours, dehydrated in a graded series of ethanol, and embedded in paraffin. Then, 5 μm thick consecutive sections were processed for HE and immunostaining by deparaffinisation with xylene and rehydration with graded ethanol.

To augment the expression of antigen in tissues, citrate buffer solution was added to the samples and they were boiled in a microwave oven. The samples were treated with a 3% hydrogen peroxide solution for 10 min to suppress the endogenous peroxidase activity and then rinsed with phosphate buffered saline (PBS). To prevent non-specific immune reactions, the samples were treated with 3% normal goat serum for 10 min, and rinsed with PBS. The primary polyclonal rabbit anti-mouse CCL22 antibody (Santa Cruz Biotechnology, Santa Cruz, CA, USA) and rabbit anti-mouse CCR4 antibody (Abcam, Cambridge, UK) were diluted 1:100 using goat serum and incubated at room temperature for 1 hour. After three 2 min washes with PBS, the sections were incubated with a biotinylated goat secondary antibody for 30 min (DAKO, Carpinteria, CA, USA). After three 2 min washes with PBS, streptavidin -horseradish peroxidase (DAKO) was added to the section for 30 min, followed by another three 2 min washes with PBS. The samples were developed with 3,3′-diaminobenzidine substrate (Vector Laboratories, Burlington, Ontario, Canada) for 1 min and counterstained with Mayer’s haematoxylin. The slides were dehydrated following a standard procedure and sealed with coverslips. The negative control was prepared by the same procedure using PBS instead of the primary antibody. Normal stomach tissues were used as the positive control.

### Immunocytochemistry staining of CCR4

Gastric cancer cells were plated in chamber slides coated with poly-L-lysine, allowed to attach for 48 hours and then used for immunocytochemistry. The cells were fixed with 0.3% H_2_O_2_ in methanol for 60 min at room temperature, washed 4 times in PBS, blocked with 3% BSA and permeabilised with PBS containing 0.1% Triton X-100 for 60 min at room temperature. The antibody, FITC-conjugated anti-mouse CCR4 polyclonal antibody (1 μg/ml, eBioscience), was incubated for 60 min at 37°C, washed 4 times and inspected under a Fluorescence Microscope (BX-51 TR32000, Olympus).

### Immunofluorescent labelling of macrophages in omental milky spots

For immunohistochemistry, the omentum was fixed in formalin for 60 min and washed three times with phosphate-buffered saline. The omentum was incubated for 60 min at 37°C with a FITC-conjugated anti-mouse F4/80 murine macrophage marker (1 μg/ml; Biolegend), washed three times in phosphate-buffered saline and then air-dried in a dark room for 12 hours. Immunohistochemical staining was directly scored and images were captured using a fluorescence microscope (BX-51 TR32000; Olympus).

### Reverse transcription–PCR analysis

Total RNA was extracted and purified from cultured MFCs using an RNeasy Mini Kit (Qiagen, Milan, Italy). The extraction include a DNase I digestion step. RNA quantity and quality was assessed by UV spectrophotometry. The RNA was transcribed into cDNA using the SuperScript First-Strand Synthesis System (Invitrogen). RT–PCR was performed with SuperScript One-Step (Invitrogen). The primers used were as follows: CCR4 sense primer 5′-GGGGTCATCACCAGTTTG-3′, CCR4 antisense primer 5′-TCTTCACCGCCTTGTTCT-3′), GAPDH forward primer 5′-CCACCCATGGCAAATTCCCATGGCA-3′, and GAPDH reverse primer 5′-TCTAGACGGCAGGTCAGGTCCACC-3′.

### Western blot analysis

Cells were washed with phosphate-buffered saline (PBS) and lysed in radioimmunoprecipitation assay (RIPA) lysis buffer (150 mM NaCl, 50 mM Tris–HCl, 0.1% sodium dodecyl sulphate, 1% Nonidet P-40, 1 mM phenylmethylsulfonyl fluoride). The lysates were cleared by centrifugation (15,000 rpm for 5 min) and the protein concentrations were determined using the bicinchoninic acid method before storage at −80°C. Equivalent amounts of protein were separated on SDS-polyacrylamide gel electrophoresis (PAGE) and transferred to polyvinylidene difluoride membranes. Membranes were blocked in 5% fat-free milk in Tris-buffered saline with 0.1% Tween 20 and incubated with the primary antibody overnight at 4°C. Immune complexes were then detected using the enhanced chemiluminescence system (Amersham, Buckinghamshire, UK).

### Cell proliferation assay

MFCs were seeded into 96-well plates at a density of 1×10^4^ cells per well (200 mL) and incubated in complete medium for 24 hours. The medium was replaced with serum-free medium containing various concentrations of CCL22, with RPMI 1640 serving as the negative control. After incubating for 24 hours, 20 μL of MTT Reagent (5 mg/mL; Sigma-Aldrich Co.) was added and incubated for 4 hours. Then, 100 μL of detergent reagent was added, left at room temperature in the dark for 2 hours and the absorbance at 492 nm recorded.

### Migration assay

Cell migration assays were performed in 6.4 mm diameter chambers with 8-mm pore filters (Becton Dickinson Labware, Franklin Lakes, NJ). MFCs were placed in the upper chamber (100 cells per well) while the lower chamber was filled with DMEM containing various concentrations of CCL22 (0, 1, 10, and 100 ng/mL). Cells were incubated for 24 hours at 37°C in 5% CO2. The cells that did not pass through the membrane pores were removed. Migrated cells were fixed and stained. Cell numbers in the lower chamber were counted in 10 random fields (×200) and expressed as the average number of cells per field of view. The data were represented as the average of three independent experiments.

## Results

### Morphology of the milky spots

**Figure 1:**
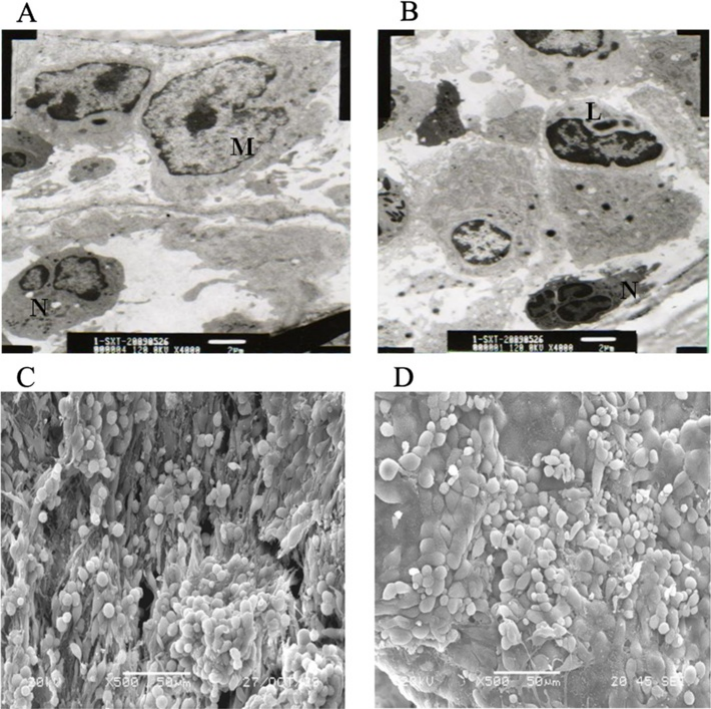
**Transmission and scanning electron microscopy of omental milky spots. (A)** Electron microscopy revealed that the cell composition of the milky spots was largely composed of abundant macrophages (M). **(B)** Some lymphocytes (L) and neutrophils (N) were also noted (magnification, 4,000X). **(C)** In the milky spot areas, the surface layer cells consisted of macrophages, lymphocytes and discontinuous mesothelial cells and were separated by intercellular gaps or pores. **(D)** In the non-milky spot areas, intercellular gaps or pores were not observed (magnification, 500X).

Electron microscopy revealed that the milky spots were largely composed of abundant macrophages (Figure 1A), with some lymphocytes, neutrophils and various stromal cells (Figure 1B).

Scanning electron microscopy revealed that the surface of these milky spots was morphologically distinct from that of the non-milky spot areas of the omentum. The surface layer cells consisted of macrophages, lymphocytes and discontinuous mesothelial cells, which were separated by intercellular gaps or pores (Figure 1C). In the non-milky spot areas, the intercellular gaps or pores were not observed (Figure 1D).

### Immunofluorescence and HE for omental milky spots

**Figure 2:**
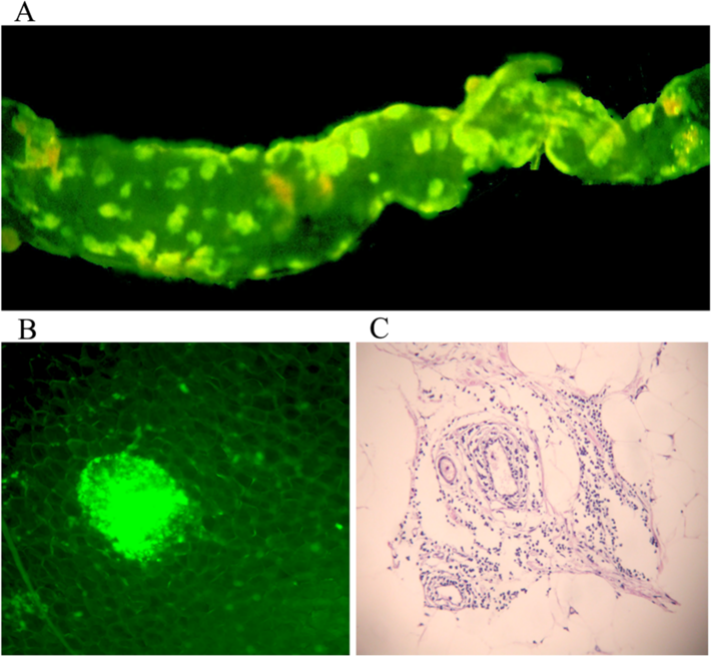
**Morphology of the milky spots. (A-B)** The greater omentum of the 615 mice was bordered by a narrow fat tissue stripe (magnification, 50X), along which cellular aggregates, known as milky spots, were observed. **(C)** Blood capillaries and arteriole vessels are found within the milky spot areas (magnification, 200X).

The whole greater omentum of the 615 mice was bordered caudally by a narrow fat tissue stripe (Figure 2A), along which cellular aggregates (green), known as milky spots, were observed (Figure 2B). Blood capillaries, arterioles and lymphocapillary vessels were found within the milky spot areas (Figure 2C).

### Visualisation of tumour cells on the omentum at early time points

**Figure 3:**
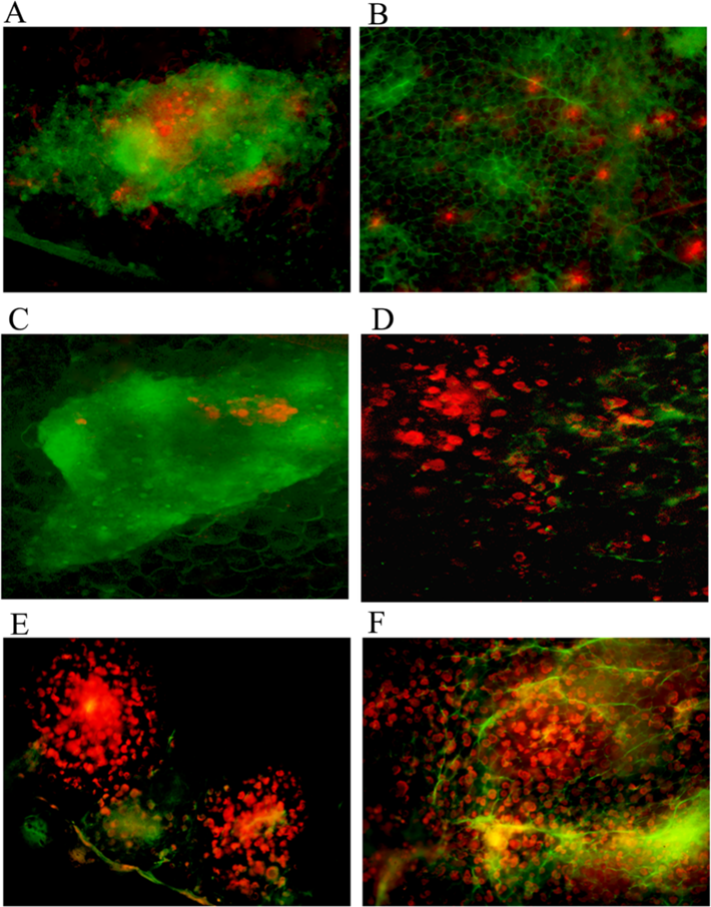
**Gastric cancer cells adhere to the omental milky spot areas at different time points. (A-B)** Image of milky spot macrophages (green) and large numbers of DiI-MFCs (red) concentrated in milky spot areas 12 h after intraperitoneal injection. **(C-D)** After 72 h, the number of DiI-MFCs decreased in milky spot areas while proliferating tumour cells in the milky spots and the formation of micrometastases were observed. Sporadic tumour cells were found in the omental non-milky spot areas. **(E-F)** Two weeks after the intraperitoneal injection, the milky spot areas were completely occupied by the proliferating gastric cancer cells and the cell cluster-type metastasis was observed. Proliferating cancer cell clusters were not observed in the non-milky spot areas and cancer cells formed single cell-type metastases. (magnification, ×200).

MFCs began to adhere to the omental milky spots at 4 hours post-injection. At 12 hours post-injection, MFCs were particularly concentrated in the milky spots (Figure 3A), while no tumour cells were found in the non-milky spot areas of the omentum (Figure 3B).

After 72 hours, proliferating tumour cells and the formation of micrometastases were noted in the milky spot areas (Figure 3C). In contrast, at 72 hours post-injection, sporadic tumour cells were found in the non-milky spot areas, while no cell clusters were detected (Figure 3D).

At 2 weeks after intraperitoneal injection, the milky spot areas were completely occupied by the proliferating gastric cancer cells and cell cluster-type metastases were observed (Figure 3E). In contrast, proliferating cancer cell clusters were not observed in the non-milky spot areas and cancer cells formed single cell-type metastasis (Figure 3F).

### CCR4 expression in gastric cancer cells

**Figure 4:**
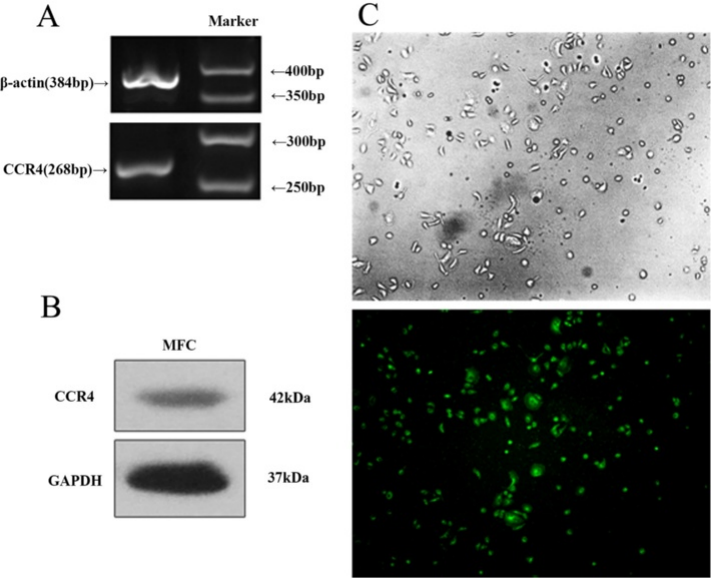
**CCR4 expression in gastric cancer cells. (A)** MFCs clearly expressed CCR4 mRNA. **(B)** Protein expression of CCR4 was also examined by Western blots. **(C)** MFCs expressed CCR4 protein and its was localised on the cell surface and/or in the cytoplasm of the MFCs.

MFCs clearly expressed CCR4 mRNA (Figure 4A). Protein expression of CCR4 was also examined by Western blots (Figure 4B). MFCs expressed CCR4 protein, which localised to the cell surface and/or in the cytoplasm (Figure 4C).

### The effects of CCL22 on the proliferation and invasion of MFCs

**Figure 5:**
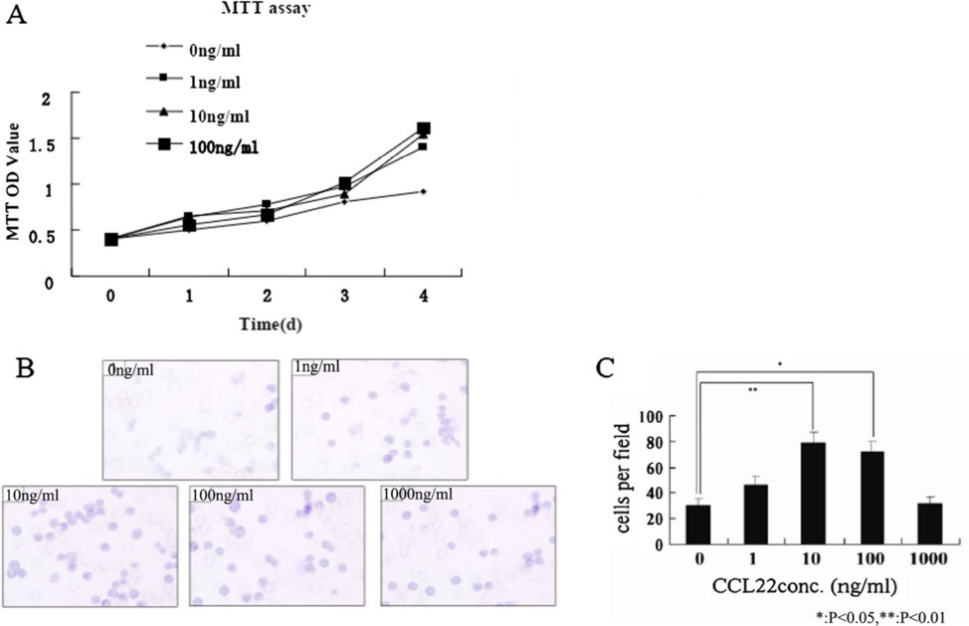
**The effects of CCL22 on the proliferation and invasion of MFCs. (A)** CCL22 significantly increased the proliferation ability at different concentrations (1–100 ng/ml) compared to the control group (P < 0.05). **(B-C)** Concentration of CCL22 between 10–100 ng/ml significantly increased migration of MFCs, with the optimal response being 10 ng/ml (P < 0.01).

The effects of CCL22 on the proliferation of MFCs were assessed by an MTT assay. CCL22 significantly increased the proliferation ability at different concentrations (1–100 ng/ml) when compared to the control group (P < 0.05) (Figure 5A).

A concentration of CCL22 between 10–100 ng/ml significantly increased migration of MFCs, with the optimal response being 10 ng/ml (P < 0.05 compared to the medium alone) (Figure 5B-C).

### CCL22 and CCR4 expression in the omental milky spot micrometastases

**Figure 6:**
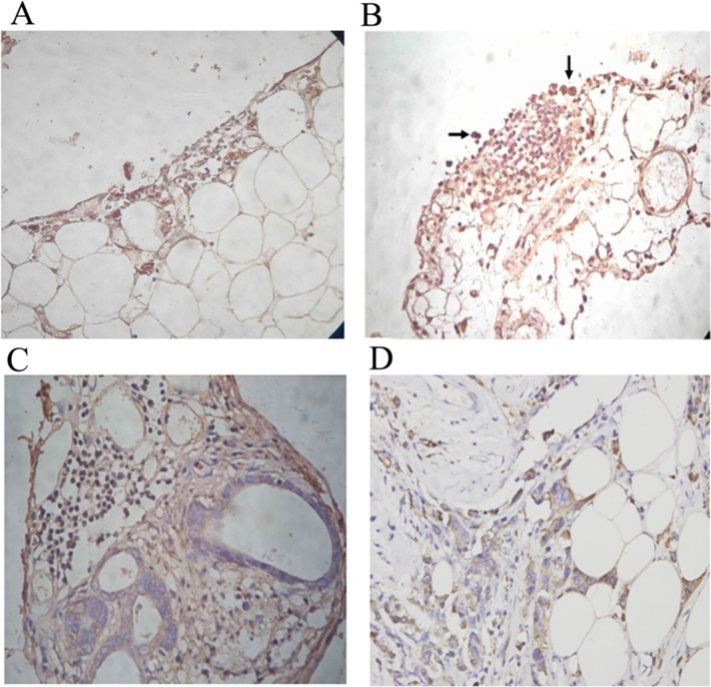
**CCL22 and CCR4 expression in the omental milky spot micrometastases. (A)** In the section of the omental milky spots, CCL22 was localised mainly on the cell surface and or in the cytoplasm of the constituent cells. **(B-D)** In the section of the omental milky spot micrometastases (12 h after intraperitoneal injection), CCR4 was localised on or in the gastric cancer cells, constituent cells of milky spot, mesothelial cells, blood cells and blood endothelial cells. **(C)** Seven days after intraperitoneal injection. **(D)** Fourteen days after intraperitoneal injection.

In the omental milky spots, CCL22 was observed mainly on the cell surface and or in the cytoplasm of the constituent cells (Figure 6A). In the omental milky spot micrometastases 12 hours, 7 days and 14 days after intraperitoneal injection, CCR4 was observed on or in the gastric cancer cells, constituent cells of the milky spot, mesothelial cells, blood cells and blood endothelial cells (Figure 5B-D).

## Discussion

The aetiology of peritoneal metastasis in gastric cancer remains to be elucidated. The release of free cancer cells from a lesion where a primary tumour invades the serosa is considered to be the origin of peritoneal metastasis [26]. More than a century has elapsed since Paget developed the theory of ‘seed and soil’ [27]. He hypothesised that certain tumour cells (seeds) selectively colonise distant organs (soil) with a favourable environment that facilitates the survival of tumour cells. In this study, we found that omental milky spots were congenial microenvironments because of their physical and chemical properties, for peritoneal free gastric cancer cells to migrate, survive, grow and form solid metastases.

Scanning electron microscopy revealed that the surface of these milky spots was morphologically distinct from that of the non-milky spot areas of the omentum. The surface layer cells consisted of macrophages, lymphocytes and discontinuous mesothelial cells and were separated by many intercellular gaps or pores. The prominent intercellular gaps or pores caused the submesothelial connective tissue and cells to be exposed to the peritoneal surface, which is proposed to represent a preferential location for tumour cell adhesion.

Micrometastases are presently classified into single-cell and small-cell cluster types [28]. The present study found a different type of tumour cell metastasis in milky spots compared to the non-milky spot areas. Two weeks after the intraperitoneal injection, the milky spot areas were completely occupied by the proliferating gastric cancer cells, and gastric cancer cells formed cell cluster-type metastases. However, proliferating cancer cells were not observed in the non-milky spot areas at the same stage, and cancer cells formed single cell-type metastases. This phenomenon can be explained by the many blood capillaries and arterial vessels present in the omental milky spot areas, which provide an adequate blood supply for the growth of gastric cancer cells.

Some studies have indicated that the process of tumour growth and metastasis involves a variety of cell-cell and cell-extracellular matrix interactions that are mediated by cell adhesion molecules. Each step requires cell adhesion molecules and receptors [29]. Mesothelial cells lining milky spots produce higher levels of cellular adhesion molecules (i.e., intercellular adhesion molecule-1) than non-milky spot areas, thus contributing to enhanced adhesion [11,12]. The role of chemokines in gastric cancer cells selectively infiltrating into the milky spots has not yet been identified. Milky spots comprise numerous macrophages that produce the chemokine MDC/CCL22 [30]. CCL22 and its’ receptor CCR4 are involved in a diverse range of pathologies. CCL22 is highly expressed in lesions created by T cell-mediated inflammatory diseases [29]. CCR4 was first reported to be preferentially expressed by Th2 cells, which are involved in humoral immunity and allergic responses [30]. CCL22 and CCR4 also play an important role in cancer growth and metastasis [17-21], thus many CCR4 receptor antagonists as well as an anti-CCR4 antibody are being developed in the pharmaceutical industry [31,32]. In this study, we found that the MFCs clearly expressed CCR4 mRNA. CCR4 protein was also examined by Western blots. CCR4 was expressed on or in the gastric cancer cells in the section of the omental milky spot micrometastases 12 hours, 7 days and 14 days after intraperitoneal injection. CCL22 was expressed mainly on the cell surface and or in the cytoplasm of the macrophages. CCL22 significantly increased the proliferation ability of gastric cancer cells, and concentrations of CCL22 between 10–100 ng/ml significantly increased migration of MFCs. Macrophages in omental milky spots not only have cytotoxic properties against tumour cells, but they also produce CCL22, which helps gastric cancer cells survive and grow into solid metastases. The MDC/CCL22-CCR4 axis plays an important role in gastric cancer cells selectively infiltrating into omental milky spots and form solid metastases.

## Conclusion

Gastric free cancer cells (seeds) locate a microenvironment containing favourable physical and chemical properties within milky spots in which they are able to survive and grow, establishing cell cluster-type metastases. The CCL22-CCR4 axis contributes to this selective infiltration.

## References

[1] Saito H, Tsujitani S, Maeda Y, Fukuda K, Yamaguchi K, Ikeguchi M, Maeta M, Kaibara N (2001). Combined resection of invaded organs in patients with T4 gastric carcinoma. Gastric Cancer.

[2] Yamashita K, Sakuramoto S, Katada N, Futawatari N, Moriya H, Hirai K, Kikuchi S, Watanabe M (2009). Diffuse type advanced gastric cancer showing dismal prognosis is characterized by deeper invasion and emerging peritoneal cancer cell: the latest comparative study to intestinal advanced gastric cancer. Hepatogastroenterology.

[3] Koenen HJ, Smit MJ, Simmelink MM, Schuurman B, Beelen RH, Meijer S (1996). Effect of intraperitoneal administration of granulocyte/macrophage-colony-stimulating factor in rats on omental milky-spot composition and tumoricidal activity in vivo and in vitro. Cancer Immunol Immunother.

[4] Berberich S, Dahne S, Schippers A, Peters T, Muller W, Kremmer E, Forster R, Pabst O (2008). Differential molecular and anatomical basis for B cell migration into the peritoneal cavity and omental milky spots. J Immunol.

[5] Mebius RE (2009). Lymphoid organs for peritoneal cavity immune response: milky spots. Immunity.

[6] Rangel-Moreno J, Moyron-Quiroz JE, Carragher DM, Kusser K, Hartson L, Moquin A, Randall TD (2009). Omental milky spots develop in the absence of lymphoid tissue-inducer cells and support B and T cell responses to peritoneal antigens. Immunity.

[7] Ikehara Y, Shiuchi N, Kabata-Ikehara S, Nakanishi H, Yokoyama N, Takagi H, Nagata T, Koide Y, Kuzushima K, Takahashi T, Tsujimura K, Kojima N (2008). Effective induction of anti-tumor immune responses with oligomannose-coated liposome targeting to intraperitoneal phagocytic cells. Cancer Lett.

[8] Abe H, Ina K, Kitamura H, Sumiyoshi H, Tatsukawa S, Yoshioka H, Fujikura Y (2009). Role of the CXCL12/CXCR4 axis in milky spots of rats bearing ascitic-type hepatoma. Anat Sci Int.

[9] Oosterling SJ, van der Bij GJ, Bogels M, van der Sijp JR, Beelen RH, Meijer S, van Egmond M (2006). Insufficient ability of omental milky spots to prevent peritoneal tumor outgrowth supports omentectomy in minimal residual disease. Cancer Immunol Immunother.

[10] Tsujimoto H, Hagiwara A, Shimotsuma M, Sakakura C, Osaki K, Sasaki S, Ohyama T, Ohgaki M, Imanishi T, Yamazaki J, Takahashi T (1996). Role of milky spots as selective implantation sites for malignant cells in peritoneal dissemination in mice. J Cancer Res Clin Oncol.

[11] van Rossen ME, Hofland LJ, van den Tol MP, van Koetsveld PM, Jeekel J, Marquet RL, van Eijck CH (2001). Effect of inflammatory cytokines and growth factors on tumour cell adhesion to the peritoneum. J Pathol.

[12] Cui L, Johkura K, Liang Y, Teng R, Ogiwara N, Okouchi Y, Asanuma K, Sasaki K (2002). Biodefense function of omental milky spots through cell adhesion molecules and leukocyte proliferation. Cell Tissue Res.

[13] Zlotnik A, Yoshie O (2000). Chemokines: a new classification system and their role in immunity. Immunity.

[14] Sallusto F, Mackay CR, Lanzavecchia A (2000). The role of chemokine receptors in primary, effector, and memory immune responses. Annu Rev Immunol.

[15] Balkwill F, Mantovani A (2001). Inflammation and cancer: back to Virchow?. Lancet.

[16] Muller A, Homey B, Soto H, Ge N, Catron D, Buchanan ME, McClanahan T, Murphy E, Yuan W, Wagner SN, Barrera JL, Mohar A, Verastegui E, Zlotnik A (2001). Involvement of chemokine receptors in breast cancer metastasis. Nature.

[17] Ishida T, Ueda R (2006). CCR4 as a novel molecular target for immunotherapy of cancer. Cancer Sci.

[18] Nakamura ES, Koizumi K, Kobayashi M, Saitoh Y, Arita Y, Nakayama T, Sakurai H, Yoshie O, Saiki I (2006). RANKL-induced CCL22/macrophage-derived chemokine produced from osteoclasts potentially promotes the bone metastasis of lung cancer expressing its receptor CCR4. Clin Exp Metastasis.

[19] Gobert M, Treilleux I, Bendriss-Vermare N, Bachelot T, Goddard-Leon S, Arfi V, Biota C, Doffin AC, Durand I, Olive D, Perez S, Pasqual N, Faure C, Ray-Coquard I, Puisieux A, Caux C, Blay JY, Ménétrier-Caux C (2009). Regulatory T cells recruited through CCL22/CCR4 are selectively activated in lymphoid infiltrates surrounding primary breast tumors and lead to an adverse clinical outcome. Cancer Res.

[20] Lee JH, Cho YS, Lee JY, Kook MC, Park JW, Nam BH, Bae JM (2009). The chemokine receptor CCR4 is expressed and associated with a poor prognosis in patients with gastric cancer. Ann Surg.

[21] Li JY, Ou ZL, Yu SJ, Gu XL, Yang C, Chen AX, Di GH, Shen ZZ, Shao ZM (2012). The chemokine receptor CCR4 promotes tumor growth and lung metastasis in breast cancer. Breast Cancer Res Treat.

[22] Rubie C, Frick VO, Wagner M, Rau B, Weber C, Kruse B, Kempf K, Tilton B, Konig J, Schilling M (2006). Enhanced expression and clinical significance of CC-chemokine MIP-3 alpha in hepatocellular carcinoma. Scand J Immunol.

[23] Yasumoto K, Koizumi K, Kawashima A, Saitoh Y, Arita Y, Shinohara K, Minami T, Nakayama T, Sakurai H, Takahashi Y, Yoshie O, Saiki I (2006). Role of the CXCL12/CXCR4 axis in peritoneal carcinomatosis of gastric cancer. Cancer Res.

[24] Yasumoto K, Yamada T, Kawashima A, Wang W, Li Q, Donev IS, Tacheuchi S, Mouri H, Yamashita K, Ohtsubo K, Yano S (2011). The EGFR ligands amphiregulin and heparin-binding egf-like growth factor promote peritoneal carcinomatosis in CXCR4-expressing gastric cancer. Clin Cancer Res.

[25] Koizumi K, Kato S, Sakurai H, Hashimoto I, Yasumoto K, Saiki I (2012). Therapeutics target of CXCR4 and its downstream in peritoneal carcinomatosis of gastric cancer. Front Biosci (Schol Ed).

[26] Tanaka T, Kumagai K, Shimizu K, Masuo K, Yamagata K (2000). Peritoneal metastasis in gastric cancer with particular reference to lymphatic advancement; extranodal invasion is a significant risk factor for peritoneal metastasis. J Surg Oncol.

[27] Fokas E, Engenhart-Cabillic R, Daniilidis K, Rose F, An HX (2007). Metastasis: the seed and soil theory gains identity. Cancer Metastasis Rev.

[28] Yanagita S, Natsugoe S, Uenosono Y, Kozono T, Ehi K, Arigami T, Arima H, Ishigami S, Aikou T (2008). Sentinel node micrometastases have high proliferative potential in gastric cancer. J Surg Res.

[29] Vulcano M, Albanesi C, Stoppacciaro A, Bagnati R, D’Amico G, Struyf S, Transidico P, Bonecchi R, Del Prete A, Allavena P, Ruco LP, Chiabrando C, Girolomoni G, Mantovani A, Sozzani S (2001). Dendritic cells as a major source of macrophage-derived chemokine/CCL22 in vitro and in vivo. Eur J Immunol.

[30] D’Ambrosio D, Iellem A, Bonecchi R, Mazzeo D, Sozzani S, Mantovani A, Sinigaglia F (1998). Selective up-regulation of chemokine receptors CCR4 and CCR8 upon activation of polarized human type 2 Th cells. J Immunol.

[31] Purandare AV, Somerville JE (2006). Antagonists of CCR4 as immunomodulatory agents. Curr Top Med Chem.

[32] Purandare AV, Wan H, Gao A, Somerville J, Burke C, Vaccaro W, Yang X, McIntyre KW, Poss MA (2006). Optimization of CCR4 antagonists: side-chain exploration. Bioorg Med Chem Lett.

